# Modeling *de novo* granulation of anaerobic sludge

**DOI:** 10.1186/s12918-017-0443-z

**Published:** 2017-07-17

**Authors:** Anna Doloman, Honey Varghese, Charles D. Miller, Nicholas S. Flann

**Affiliations:** 1 Department of Biological Engineering, Utah State University, Old Main Hill 4105, Logan, 84322-4105 UT USA; 2 Department of Computer Science, Utah State University, Old Main Hill 420, Logan, 84322-4205 UT USA

**Keywords:** Agent-based modeling, Multicellular modeling, Anaerobic granulation, Wastewater treatment

## Abstract

**Background:**

A unique combination of mechanical, physiochemical and biological forces influences granulation during processes of anaerobic digestion. Understanding this process requires a systems biology approach due to the need to consider not just single-cell metabolic processes, but also the multicellular organization and development of the granule.

**Results:**

In this computational experiment, we address the role that physiochemical and biological processes play in granulation and provide a literature-validated working model of anaerobic granule *de novo* formation. The agent-based model developed in a *cDynoMiCs* simulation environment successfully demonstrated a *de novo* granulation in a glucose fed system, with the average specific methanogenic activity of 1.11 ml *C* *H*_4_/g biomass and formation of a 0.5 mm mature granule in 33 days. The simulated granules exhibit experimental observations of radial stratification: a central dead core surrounded by methanogens then encased in acidogens. Practical application of the granulation model was assessed on the anaerobic digestion of low-strength wastewater by measuring the changes in methane yield as experimental configuration parameters were systematically searched.

**Conclusions:**

In the model, the emergence of multicellular organization of anaerobic granules from randomly mixed population of methanogens and acidogens was observed and validated. The model of anaerobic *de novo* granulation can be used to predict the morphology of the anaerobic granules in a alternative substrates of interest and to estimate methane potential of the resulting microbial consortia. The study demonstrates a successful integration of a systems biology approach to model multicellular systems with the engineering of an efficient anaerobic digestion system.

## Background

**Fig. 1:**
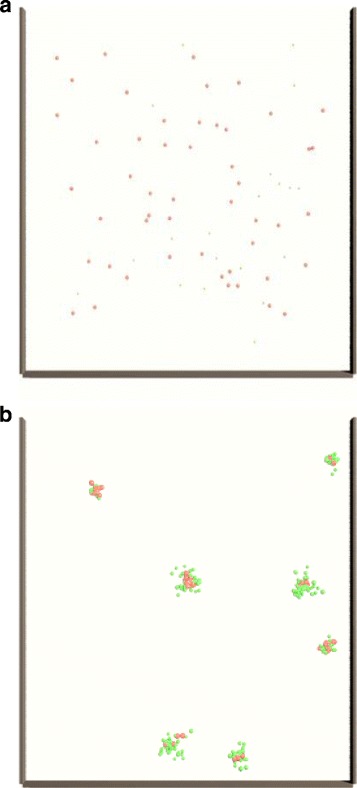
Reactor scale model. **a** initial random distribution of two types of cells in a UASB-like environment; **b** formation of cell aggregates due to the mechanical forces, mutual adhesion and random agitation in the UASB-like environment

An efficient anaerobic digestion (AD) of organic matter is a result of a complex microbial interaction inside a bioreactor. For the high-rate anaerobic digestion of a feedstock, an up-flow anaerobic sludge blanket reactor (UASB) is a common choice. The superior performance of this reactor is due to the particular organization of microorganisms into spherical granular structures. The process of granulation was first noticed and documented in the early 1980s [1, 2] and since then a number of anaerobic granulation theories have been presented. The main reasoning for the granulation *per se* is the up-flow velocity inside sludge bed of a UASB reactor. Microbial cells moving up with the flow of the feed tend to stick to the other microbial cells. Such sticking behavior prevents a washout of the microbial inoculum from a reactor since the outlet for the digested feed is located in the top of the reactor [3, 4] (see Fig. 1). The most widely accepted theory states that granulation starts with a formation of a future granule’s core, comprised of filamentous methanogenic bacteria *Methanothrix*, together with *Methanosarcina*, which secrete extracellular polymers (ECP) [5–7]. The surface charge of this core changes and become attractive for the oppositely charged anaerobic bacteria that are present in the dispersed inoculum of a UASB rector [8–10]. Chemo-attractance of other bacteria towards ECPs and substrate around the granule core may also play a major role in the further aggregation and formation of mature granules [11, 12]. Despite these possible explanations of the granulation process, there is still no agreement on which of the possible theories correctly explain this most important and crucial role of granulation. The key factors of granulation are still to be determined, whether they are physical, biochemical or a combination of physicochemical properties of the cells and the way the organic matter transforms over space and time.

An effective means to get a better understanding the granulation process is through the construction of a computational granulation model. This model must incorporate testing of different key granulation factors. There are already some granulation models available in the literature, but they do not describe a process of *de novo* granulation and only describe the kinetics of anaerobic digestion with an already mature granular consortia. For example, one of the earliest models [13] assumes a layered granule structure with a homogeneous distribution of microbial groups from the very beginning of the simulation. Authors describe the kinetics of substrate transformation in a mature granule that reached a steady state. Using the same assumption [14] they successfully predicted the substrate distribution inside a granule, based on diffusivity gradient inside a biomass. Authors of another study [15] took the substrate kinetics in the granule one step further, incorporating behavior of granular agglomerates into the operation predictions of the whole UASB reactor. The mass of granules in a reactor, rates of granule decline and general bacterial growth kinetics were used as a basis for the model. In another study [16], researchers have applied a cellular automata theory, developed by Wimpenny et al., [17], to model granulation during anaerobic digestion. However, authors assumed a homogeneous layered structure of a granule and obtained calculated values of substrate utilization rates that do not agree with the experimental data they used as a reference.

A commonly applied assumption of a homogenous-layered structure of anaerobic granule does not conform with experimental data. In particular, data suggests a spatially organized granule containing a mixed composition of bacterial groups inside the granule. In models lacking this property, there is no strict compartmentalization of trophic groups, like methanogens and acidogens, in the core and outer layer, respectively. Strict anaerobes, like methanogens, can also be found in the outer layer of the granule, as visualized with fluorescent probing experiments and scanning electron microscopy [18–21]. A non-homogeneous bacterial distribution is investigated in a model described in [22]. However, the study does not address the process of granulation itself, and an entirely formed granule is employed as an initial condition and seed of a model. The model, therefore, predicts a mature granule’s further development, growth, and formation of an inert core insie it.

An enormous amount of knowledge has been developed on predicting the rates of anaerobic digestion in UASB reactors with mature granules. However, these models are not complete and do not represent the actual input for large scale applications, specifically those of the widely accepted biochemical model of the anaerobic digestion process (ADM1) [23]. The most recent review of a current status of ADM1 clearly states the need to thoroughly address the application of ADM1 to various types of anaerobic reactors, UASB in particular. Thus, a complete and trustful model of anaerobic digestion in UASB must take into account both granulation in general and initial *de novo* granulation [24]. Knowledge of the critical parameters facilitating *de novo* granule formation will aid in robust UASB reactor operation and production of increased methane yields with high organic matter transformation rates.

To model *de novo* anaerobic granulation, a number of computational platforms has been reviewed to find the best fit. The cellular Potts model was a pioneer [25] in biofilm modeling and has been extensively implemented in modeling of biofilms of the eukaryotic origin [26, 27]. To effectively apply this approach to the microbial liquid-based environment (thus without influence of attachment/detachment to the substratum), this model needs a lot of improvements, to prevent formation of artifacts [28, 29]. To model *de novo* anaerobic granulation, a number of computational platforms has been reviewed to find the best fit. The cellular Potts model was a pioneer [25] in biofilm modeling and has been extensively implemented in modeling of biofilms of the eukaryotic origin [26, 27]. To effectively apply this approach to the microbial liquid-based environment (thus without influence of attachment/detachment to the substratum), this model needs a lot of improvements, to prevent formation of artifacts [28, 29]. A simulator framework *cDynoMics* [30, 31], on the other hand, is more quantitative and is very flexible to adjust for modeling of bacterial aggregates. This framework has built-in functions to specify all the necessary substrate limiting kinetics for cell growth and biomass decay due to the starvation, which are absent in other previously described platforms. Absence of a solid substratum in the anaerobic digestion system excludes need for the use of attractive van der Waals force in the model, unlike in other reported biofilm developing tools [32].

A model of *de novo* granulation proposed in this paper addresses some of the key aspects that influence aggregation of microbial biomass into defined granular structures. Those key elements include: initial concentrations of the substrate used as a feedstock for anaerobic digestion; ratio of methanogenic and acidogenic cells at the start of the reactor; the role of chemotactic attractions and cell-to-cell adhesion properties. This study addresses all these factors. Additionally, an extensive computational search of the initial parameter values is made to determine an optimal initial combination that yields the highest start-up methane production rates.

## Results and discussion

Simulation experiments were conducted on the computational granulation model to give insights into different stages in the development of granules in aerobic sludge reactors. Where available, literature supported model parameters were employed. Other parameters, such as those that influence particle aggregation and mechanical sorting, were fine tuned based on correspondence between observations made from simulations and comparisons with reported granule images. The resulting granule spatial organization and product production of model simulations are analyzed and compared with values from real biological systems. Another objective of the study was to employ a search engine to find the amount of initial glucose concentration and populations of methanogens and acidogens that lead to optimal methane production.

### Study I: reactor scale model

In the reactor scale phase of modeling, randomly distributed acidogens and methanogens (illustrated in Fig. 1 a) interact with each other in a simulated UASB reactor environment, where upflow velocity and agitation play key roles to promote granulation of sludge. In the simulated environment microbial cells move around the system due to agitation and cells are bound together due to biomechanical adhesive forces, allowing formation of cell agglomerates (illustrated in Fig. 1 b).

### Study IIa: stages of granule formation

**Fig. 2:**
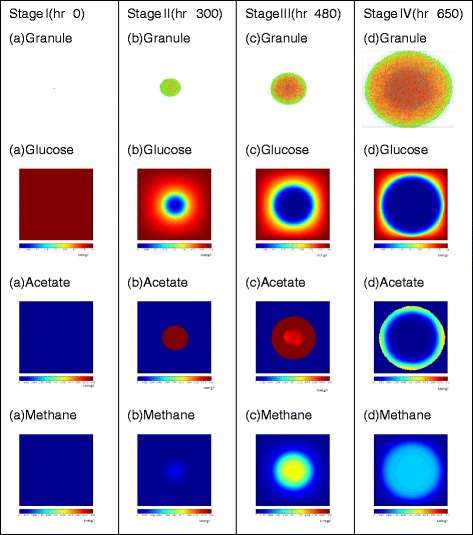
Simultion of 0.5 mm granule formation. Stages of simulated *de novo* granulation and associated dynamic changes in the solutes concentrations (glucose, acetate and methane). Only the critical time points of simulation are depicted through stages I-IV (t =0 h through t =650 h)

To investigate the development of a mature granule and dynamic changes in the cell growth, consumption of glucose, a series of simulator output snapshots were performed (Fig. 2). At the initial stage (t=0 h), single cell aggregate appear as a small cluster of acidogens and methanogens (zoomed from Reactor scale model, Fig. 1). As time proceeds (t =300, t =480 and t =700 h) cells grow and corresponding solute gradients demonstrate accumulation of acetate and methane in the system. Methane, being a volatile compound, is slowly diffused out of the system and depicted values on the scale of gradient images are not the cumulative values, as in the case of the glucose and acetate. At 480 h of granule development, a black “dead” core of cells start to emerge in the middle of the granule sphere. Appearance of a “dead” core is due to the diffusion boundaries of glucose or acetate inside granular cluster. Thus, cells of both types (acidogens and methanogens) are not getting enough energy supply and are forced to transition into the inert biomass. This transition is set to be irreversible in the model, thus leading to a formation of a “dead core”. A similar core can be seen on the Fig. 4a of the laboratory-observed granule, which is used as evaluation criterion in current study and is descried later in detail. The final stage of granule development simulation (t =650 h) demonstrates a mature granule with 0.5 mm in diameter.

### Study IIb: analysis of granule growth dynamics

**Fig. 3:**
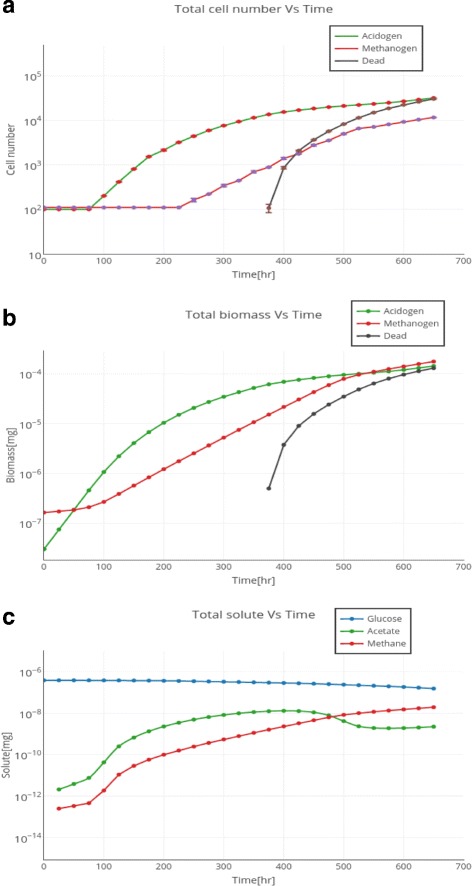
Simulation related changes in solute concentrations and cell biomass. A close-up of the dynamic changes in the **a** cell number over simulation time, **b** cell biomass over simulation time and **c** solutes concentrations over simulation time. All the changes are graphed for each type of the cell (acidogens, methanogens, inert dead type) and each type of the solute (glucose, acetate, methane). Ten simulations with different random seeds were graphed to demonstrate standard deviation in the monitored values

In addition to visual (qualitative) investigation of *de novo* granulation, a close up quantitative study was performed on dynamic changes in solute amounts and cell biomass accumulation (both in values of cell numbers and cell biomass numbers). Graphs for dynamic changes are provided in Fig. 3. Figure 3a demonstrates changes in the total number of two types of cells (acidogens and methanogens) with regard to the simulation time. Simulation was initiated with 100 cells of each type. Due to the fast growth of the acidogens (see the Table 1 with growth kinetics parameters), we can see an exponential growth of acidogens from t =80 h to t =360. A similar dynamic is depicted in Fig. 3b. Due to the product inhibition by the produced acetate and lack of diffused glucose, acidogens decrease their relative growth rate and reach the stationary phase of growth at around t =600 h. Dynamics of methanogens growth is slightly different, mainly due to the lack of available acetate from the start-up of the system and a lower growth rate, contrary to acidogens (Table 1 with model parameters). Methanogen growth goes through a long lag phase (t =0 h until t =220 h), where biomass is accumulated at a very slow rate (Fig. 3b). At this lag phase methanogen cells are waiting for the supply of acetate from acidogens. As soon as enough acetate is accumulated in the system (around t =220 h), methanogens start exponential growth and decrease their relative growth rate at about t =520 h. This decrease is in direct correspondence with the amount of available acetate in the system at the same time period (t =480–500 h), (Fig. 3c) when acidogens are inhibited by the produced acetate and are not provided with a high flow of glucose (due to the slow diffusion into the center of the granular biomass). Kinetics of acetate accumulation/conversion and methane production are in a good correlation with experimental data reported by Kalyzhnyy et al. and others [33–36].

**Fig. 4:**
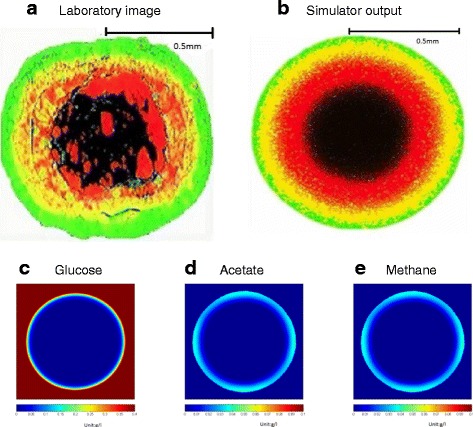
Validation of the *de novo* granulation model via qualitative analysis. **a** Laboratory image courtesy of Sekiguchi et al. (1999), where *green fluorescence* label was used for Bacteria (represented by a single group of acidogens in current study), *red fluorescence* was emitted by Archaea (represented by a single group of methanogens in current study), *yellow color* correlates with overlapped *red* and *green fluorescence* and *black color* represents absence of fluorescence hybridization, and thus, absence of cell biomass (denoted as dead core here). **b** An image of granule simulated with current model. Same color labeling of the cell types is applied. **c**, **d** and **e** Distribution of the three solutes defining simulation of granulation (glucose, acetate, methane) at the final time point (t =800 h) of the simulation

**Table 1:** Parameters used in model and their correspondent values

Parameter summary				
Model parameter	Symbol	Value	Unit	References
Solutes				
Diffusion of glucose in liquid	*D*_*g*_	5.8×10^−6^	*m* ^2^ /day	[59]
Diffusion of acetate in liquid	*D*_*a*_	1.05×10^−4^	*m* ^2^ /day	[59]
Diffusion of methane in liquid	*D*_*m*_	1.29×10^−4^	*m* ^2^ /day	[60]
Biofilm Diffusivity	*γ*	30	%	[42]
Acidogens				
Cell mass	*B*_*a*_	300	fg	[61]
Division radius		3	*μ*m	[62]
Maximum growth rate	$\hat {\mu _{a}}$	0.208	*h* ^−1^	[61], [56, 63]
Substrate saturation constant	Ks	0.26	g/L	[35, 56]
Product inhibition constant	Ki	0.1	g/L	[56, 63]
Biomass conversion rate	*α*_*bg*_	0.3	$\frac {g_{biomass}}{g_{glucose}}$	[56, 57]
Substrate conversion rate	*α*_*ag*_	0.82	$\frac {g_{acetate}}{g_{glucose}}$	[56, 63]
Death delay		48	*h*	Estimated
Death threshold		0.02	g/L	Estimated
Methanogens				
Cell mass	*B*_*m*_	1500	fg	[62]
Mass of EPS capsule		10	fg	[54]
Division radius		3	*μ*m	[62]
Maximum growth rate	$\hat {\mu _{m}}$	0.1	*h* ^−1^	[33, 54]
Substrate saturation constant	Ks	0.005	g/L	[54]
Biomass conversion rate	*α*_*ba*_	0.15	$ \frac {g_{biomass}}{g_{acetate}}$	[33, 35]
Substrate conversion rate	*α*_*ma*_	0.26	$\frac {g_{methane}}{g_{acetate}}$	[33]
Death delay		48	*h*	Estimated
Death threshold		0.00001	g/L	Estimated

### Study III: formation of a mature granule

Figure 4 shows images of a 1 mm in diameter granule, obtained from both a laboratory experiment reported by Sekiguchi et al. [19] (Fig. 4a) and an image from our simulated model (Fig. 4b). Simulation of 1 mm in diameter granule formation took 800 h (around 33 days), which corresponds to the published studies observing granulation in UASB reactors [20, 37]. Figure 4c, d and e depict distribution of solutes (glucose, acetate, and methane) at the final stage of simulated granule growth (t =800 h). One can note a sharp decrease in the glucose diffusion inside the granule, with regard to the biofilm diffusivity capacity. Since acetate is consumed by methanogens during their growth and converted to methane, there is a low concentration gradient of both chemicals on the final images (Fig. 4c, d, e). Overall, solute distributions for 1mm granule follow a similar pattern as for the 0.5 mm granule, described earlier. Key point in conducting simulation of a 1mm granule development is to demonstrate radial growth, without substantial changes in the overall morphology. Thus, initial stages of granule formation are the key factors for granulation *per se*.

### Validation of the model

**Fig. 5:**
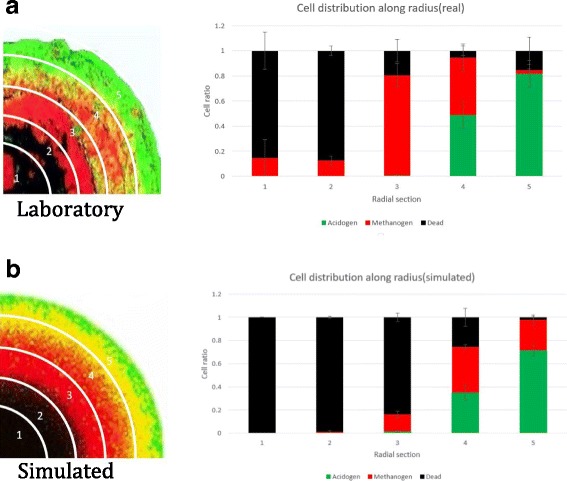
Validation of the *de novo* granulation model via quantitative analysis. Validation was done via analysis of the three cell type radial distribution in the both laboratory (**a**) and simulated granules (**b**). Both granules were divided into four quarters and each quarter was analyzed for cell distribution. Differences in the cell numbers at the same radial distance in four quarters are depicted in a form of standard deviation. *Red*, *green* and *black colors* of the bars on bar chart represent acidogen, methanogen and dead cells respectively

Validation of the model performance was conducted both qualitatively (Fig. 4a, b) and quantitatively (Fig. 5). Visual comparison of a published fluorescent-labeled image of granule with simulated granule image demonstrates a striking similarity in spatial distribution of main trophic groups of microorganisms - acidogens, methanogens and “dead” biomass. Irregularities and hollow parts (black color) in the published granule image (Fig. 4a) are possibly caused by the upflow velocity of the liquid and particulate matter in a UASB reactor, where the granule was developed [19], which might have damaged spherical shape of the immature granule, causing mature granule to change its shape and grow further with hollow compartments. Another possible explanation might be granule division. It is well documented [8–10] that due to the shear stress in a UASB reactor, granules cannot grow uncontrollably and will eventually split into “daughter” granules. Those “daughter” granules are susceptible to attachments of additional microbial cells, floating in UASB sludge bed. Those newly attached cells might cause irregularities in future mature granules in forms of randomly distributed cell clusters in a presumably inert (“dead”) core (red-labeled cell clusters on Fig. 4a). To validate our simulated model quantitatively, we conducted image processing of the published data and used an algorithm to count the number of distinctly colored pixels/cells at the different distances from the center of the granule image (Fig. 5). We used 4 quarters of a spherical granule in the analysis to provide standard deviations of spatial distribution of three distinct cell groups – acidogens, methanogens and inert (“dead”) biomass. Results of quantitative distribution of three main cell types in both simulated and real images are in a good correlation, accept for the radial section “3”. Such slight discrepancy is due to the possible “division to daughter granules” history of the laboratory granule.

### Parameter scan for optimized methane production

**Fig. 6:**
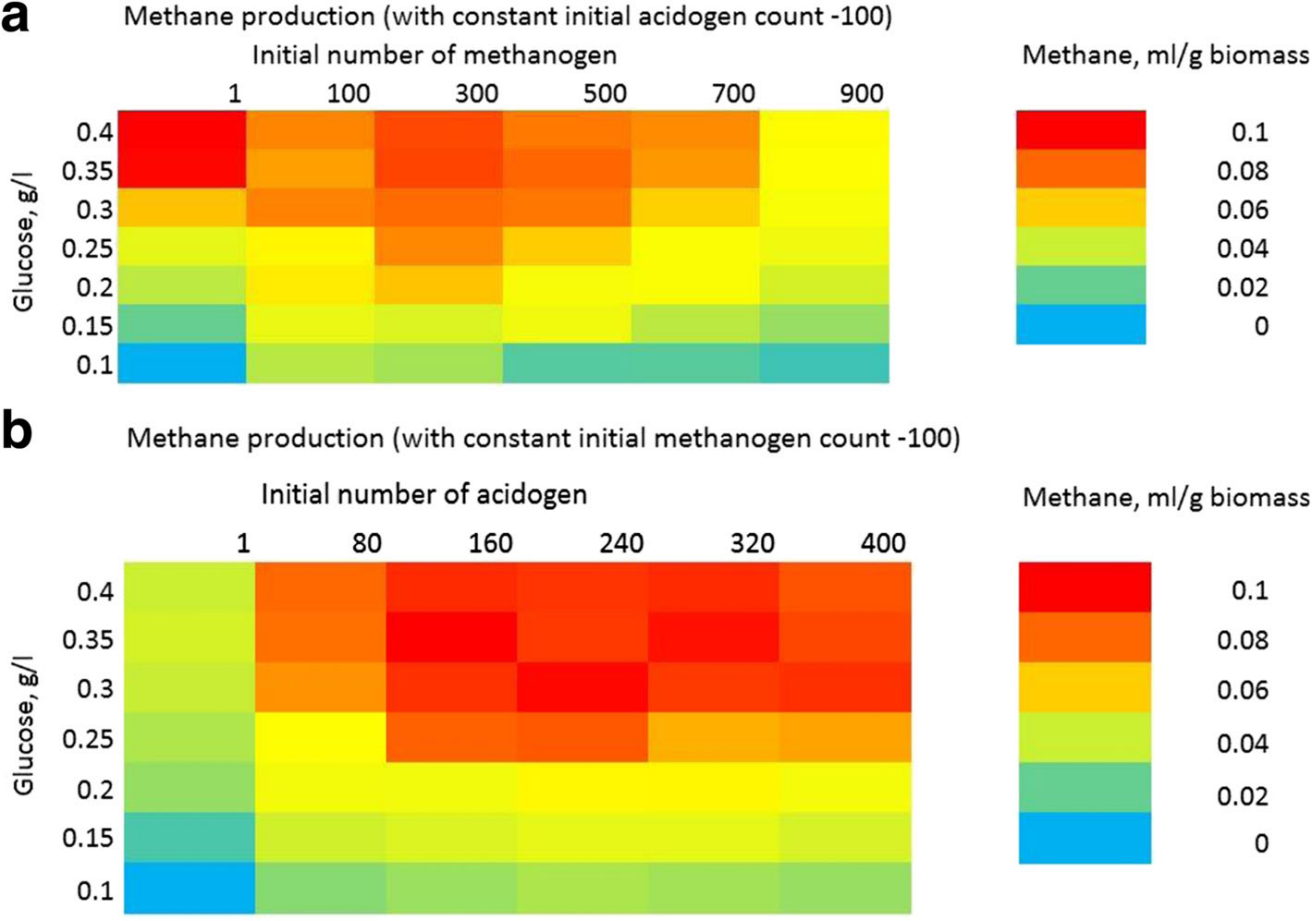
Parameter scan for the methane production in simulated granule. Parameter scan for the methane production in simulated granule with **a** varying initial number of methanogen cells (constant initial acidogen cell count) and **b** varying initial number of acidogen cells (constant initial methanogen cell count). *Red color* of the heatmap section has the highest value of methane produced (in milliliters of methane per gram of biomass), while *blue* heatmap section has the lowest value of produced methane. Parameter scan was conducted for 0.5 mm granule size and for the period of 650 simulation hours

Main objective of the parameter scan is to estimate a combination of cell ratio (acidogens:methanogens) and glucose supply needed to start anaerobic system to achieve a desired (maximum) methane yield. The corresponding protocol parameter for glucose value is “SBulk” in world section. The “init area number” for acidogens and methanogens in the species section is used to determine the initial cell ratio for the simulations. The minimum and maximum value of the interval in which the search should be performed is given as an input to the search engine. The methane productivity (calculated from the solute concentration file output from simulator) is given as fitness function for the engine. The search engine simulated granule formation for several combinations of parameter values within the input interval and calculated total methane produced. The result is produced as a heatmap in Fig. 6.

Figure 6 depicts amount of methane produced (in milliliters) per gram of biomass with varying amount of glucose supplied initially into the system (0.1 to 0.4 g/l). Figure 6a has a constant initial acidogen count of 100 cells, and heatmap demonstrates varying amounts of methane produced with different glucose concentrations and different numbers of initial methanogen cells (from 1 to 900 cells). Same scheme is followed on Fig. 6b, but with varying initial numbers of acidogens (from 1 to 400) and constant initial methanogen count of 100 cells.

One can note from both Fig. 6a and b that increased amount of glucose correlates with increased amount of methane produced in the system. Also, in general increased number of starting cells of acidogens (Fig. 6b) let to the higher amounts of methane produced. This correlates with the earlier explored kinetics of methanogen/acidogen growth, when methanogens are waiting for acetate supply until they start to grow and produce methane. Parameter scan also helped to identify an important observation that a ratio of methanogen cells to acidogens should not be in a high favor of methanogens (100 acidogens and 900 methanogens on Fig. 6a), since this leads to a decreased amount of methane production. The reason for such correlation is lack of acetate in the system to support growth of such a big number of methanogenic cells, which are forced to starve and die off.

## Conclusions

A model of anaerobic granulation from digestion of glucose to methane has been successfully implemented in an agent-based simulator framework, *cDynoMiCs*. Simulation studies incorporated modeling of both reactor and single agglomerate scale granule development. Utilized growth mechanisms for generalized glucose-consuming/acetate-producing bacteria and acetate-consuming/methane-producing bacteria resulted in a well-correlated kinetic patterns of substrate conversions and biomass growth (Fig. 3). We were able to successfully qualitatively and quantitatively validate the architecture of the developed simulated anaerobic granule with the granule images and cell distribution from experimental literature studies (Figs. 4 and 5). The described granulation model has direct applications for designs of experiments, to predict yields of methane gas from substrates of interest. One application of the model was successfully demonstrated in this paper via parameter scan algorithm, searching through different acidogens:methanogens cell ratios and glucose feed that is needed to start anaerobic system to achieve a desired (maximum) methane yield. By changing the parameters of microbial growth to fit bacteria of a specific interest (the bacteria one is targeting to explore in an AD experiment), researchers can apply this model to predict efficiencies of anaerobic digestion in a system. The tested parameter scan is directly applicable to the studies with low-strength feed streams to UASB reactors, such as AD of brewery wastewater (COD =100-800 mg/L) [38], some municipal and industrial wastewaters (COD =100-400 mg/L) [39, 40] and effluents from petroleum refineries (COD from 68 mg/L) [41]. Further development of the model will include a parameter search to investigate methane production from medium and high strength wastewaters. The current model of anaerobic granulation and methane production from simple feed sources (glucose) can be expanded to accommodate microbial conversion of more substrates, such as a mixture and proteins and carbohydrates. This expansion will make it possible to study granulation and methane potential from a more realistic scenario of wastewater feed, such as dairy and municipal wastewaters. A granulation model from a complex feed should result in a less stratified granule, due to the differential diffusions of the main feed components and a more complex patterns of microbial growth kinetics [18].

In addition, a model framework (*iDynoMiCs*) can be further modified to simulate detachment of excessive biomass from granular surface (simulating sheer stress described in the UASB reactor environment [4, 42–44]) and breakage of a granule into daughter clusters, that subsequently give rise to mature granules with a more complex morphology [18, 21, 45]. Since current model assumes spherical types of cells, exploration of filamentous type of methanogenic bacteria influencing *de novo* granulation based on the “spaghetti theory” is something of future interest [32, 46]. Another possible realm to expand development and application of current granulation model is to explore the mechanisms of enhancing anaerobic granulation, such as addition of positively charged ions and particles of polymers into the UASB system [47, 48]. To converge granulation model with reactor-like environment, a *Biocellion* modelling environment can be used [49, 50]. Possibility to parallelize computation load in Biocellion would eliminate the main bottleneck of the *cDynoMics* and allow development of a whole reactor model with simultaneous substrate conversion and anaerobic granule development. The current model of the *de novo* anaerobic granulation and its immediate applications will aid future discoveries in the field of anaerobic digestion, which is regaining its value and popularity in sustainable energy.

## Methods

The process of granulation is modeled at two spatial scales in the simulation. At the macroscale, the reactor process is simulated where the cells are introduced into an agitated system (due to the upflow velocity in UASB reactor), cells interact and form multiple agglomerates (centers of granulation). At the mesoscale, simulations are performed that focus on the growth and development of one such agglomerate into a mature granule.

In the macroscale, randomly distributed acidogenic (further referred to as “acidogens”) and methanogenic cells (further referred to as “methanogens”) are introduced into random positions within the reactor. The particles experience mechanical forces due to agitation in the system as well as biomechanical forces due to homogeneous and heterogeneous adhesion and formation of EPS-driven interactions. As a cumulative effect of these forces, cells come close to each other and form several agglomerates.

To closely monitor the growth patterns in the formation of a granule, the mesoscale simulation is designed to focus on the development of a single granule (from the initial agglomerate of acidogens and methanogens formed during the macro studies). In UASB bioreactors, granules move freely in an agitated system, where the supplied solutes are relatively mixed. To simulate such a mixed environment for the granule growth, we provide a continuous supply of one solute (glucose) from all the sides of the simulation domain with diffusivity as defined in Table 1. The model executes growth reactions that represent the consumption of the supplied glucose by the acidogens, the secretion of the acetate as a metabolite of acidogens and the consumption of acetate by methanogens, which is converted into the methane gas.

An agent-based simulator framework, *cDynoMiCs* [31] is used in this experiment. *cDynoMiCs* is an extension of *iDynoMiCs* framework developed by the Kreft group at University of Birmingham [51] specifically for modeling biofilms. *cDynoMiCs* includes eucaryotic cell modeling processes with the addition of extracellular matrix and cellular mechanisms such as tight junctions and chemotaxis. Each cell is represented as a spherical particle, which has a particular biomass, and implements type and species-specific mechanisms to reproduce cellular physiology. Biochemically, particles can secrete or uptake chemicals that are diffused through the domain by executing reactions. Biomechanically, particles exhibit homogeneous and heterogeneous adhesion, and the formation of tight junctions. Particles model growth by increasing their biomass according to metabolic reactions and split into two particles once a maximum radius threshold is reached. They can also switch from one type of particle to another based on specific microenvironmental conditions and internal states. The simulation process interleaves biomechanical stress relaxation where the particles are moved in response to individual forces, along with the resolution of biochemical processes such as secretion, uptake, and diffusion by a differential equation solver. We assume that the solute fields are in a pseudo steady-state with respect to biomass growth [51].

Particle growth and division can cause particles to overlap, creating biomechanical stress. To resolve this problem a process called shoving is implemented. When the distance between two particles is less than a fixed threshold set by the particle size, a repulsive force is generated to push them apart, proportional to the overlap distance between the two particles. Then the relaxation process commences that iteratively moves each particle in response to its net force, then recalculates the forces due to the movement. The process terminates when only negligible forces remain, and the system has reached a pseudo steady state.

*cDynoMiCs* adds new functionality to the Java code of *iDynoMiCS* and extends the XML protocol, used to specify many different types of simulations. *iDynoMiCS* writes plain-text XML files as output, and these may be processed using any number of software tools, such as Matlab and R. In addition to XML files, *iDynoMiCS* also writes files for POV-Ray that is used to render 3-D ray-traced images of the simulation. For the experiment to form the 1mm granule a 1.16 mm × 1.16 mm domain size was used. For all other experiments, a 508 *μ*m × 508 *μ*m domain size (2D) is used. A summary of the protocol parameter values can be found in Table 1.

Three solutes glucose (*S*_*g*_), acetate (*S*_*a*_) and methane (*S*_*m*_) exist within the reactor model. The distribution of these solutes is controlled by Eqs. 1, 2, and 3 respectively. The diffusion coefficients and reaction rates take different forms for each region depending upon the spatial distribution of acidogen biomass (*B*_*a*_), methanogen biomass (*B*_*m*_) and dead biomass (*B*_*d*_) described in Eq. 4. The effective diffusion coefficient is decreased within the granule compared with the liquid value in order to account for the increased mass transfer resistance. The diffusivity values used for the model (specified in Table 1) are taken from literature related to biofilm diffusivity studies [42, 52]. The growth rate of acidogens is *μ*_*a*_(*S*_*g*_,*S*_*a*_), defined in Eq. 8, and the growth rate of methanogens is *μ*_*m*_(*S*_*a*_) defined in Eq. 9.

1 $$ \frac{\partial S_{g}}{\partial t} = B(x,y).D_{g}.\frac{\bigtriangledown^{2} S_{g}}{{\partial x}{\partial y}}- \mu_{a}(S_{g},S_{a}).\frac{B_{a}}{\alpha_{bg}}   $$

2 $$ \frac{\partial S_{a}}{\partial t} = B(x,y).D_{a}.\frac{\bigtriangledown^{2} S_{a}}{{\partial x}{\partial y}}+ \mu_{a}(S_{g},S_{a}).\frac{\alpha_{ag}.B_{a}}{\alpha_{bg}}   $$

3 $$ \frac{\partial S_{m}}{\partial t} = B(x,y).D_{m}. \frac{\bigtriangledown^{2} S_{m}}{{\partial x}{\partial y}}+\mu_{m}(S_{a}).\frac{B_{m}}{\alpha_{ba}}   $$

where,

4 $$ B(x,y)=\left\{ \begin{array}{ll} 1.0 & \text{if location~} x,y \text{~contains no biomass}\\ \gamma & \text{if location~} x,y \text{~contains biomass} \end{array} \right.   $$

Equations 5 and 6 describe acidogen and methanogen biomass changes as a function of local acetate and glucose concentration. Cell death due to lack of food is modeled using a discrete switching mechanism defined as the function *d* *i* *e*(*B*_*i*_) in the equations. Acidogen cells are converted to dead cells when the amount of glucose is below a threshold value (death threshold in Table) for a period of 48 h. Similarly, the methanogen cells are converted to dead cells when the amount of glucose is below a threshold value (death threshold in Table 1) for a period of 48 h. The rate of increase in dead cell mass is define in Eq. 7. The parameter values for controlling cell death are estimated due to the lack of studies quantifying the response of acidogen and methanogen cells to nutritional stress.

5 $$\begin{array}{*{20}l} \frac{\partial B_{a}}{\partial t} &= \mu_{a}(S_{g},S_{a}) B_{a}-die(B_{a}) \end{array} $$

6 $$\begin{array}{*{20}l} \frac{\partial B_{m}}{\partial t} &= \mu_{a}.S_{a}. B_{m}-die(B_{m}) \end{array} $$

7 $$\begin{array}{*{20}l} \frac{\partial B_{d}}{\partial t} &= die(B_{a}) + die(B_{m})  \end{array} $$

Acidogens grow by consuming glucose and producing acetate described by the Monod-kinetic Eq. 8, where $\hat {\mu }_{a}$ is the maximum growth rate for acidogens. Similarly, methanogen growth by consuming acetate and producing methane described by Monod-kinetic Eq. 9, where $\hat {\mu }_{m}$ is the maximum growth rate for mathanogens. Values for growth constants, such as biomass yield and substrate conversion rate, for both acidogens and methanogens were taken from literature and averaged. Thus, maximum growth rate for acidogens was twice as high as that that of methanogens, see [3, 35, 53–58]. Biomass decay rate is not taken into account for both cell types, since decay for anaerobic type of growth is usually less or equal to 1% of specific growth rate and thus can be ignored [58]. Non-competitive product inhibition is considered for growth of acidogens [58], but not for the methanogens, assuming low inhibition of methanogenic growth by excess amount of acetate.

8 $$ \mu_{a}(S_{g},S_{a})=\hat{\mu}_{a}.\frac{{S_{g}}}{{(K_{sg}+S_{g})}}.\frac{K_{i}} {{(K_{i}+S_{a}})}  $$

9 $$ \mu_{m}(S_{a})={\hat{\mu}_{m}}\frac{{S_{a}}}{{K_{sa}+S_{a}}}  $$
